# PS-MCL: parallel shotgun coarsened Markov clustering of protein interaction networks

**DOI:** 10.1186/s12859-019-2856-8

**Published:** 2019-07-24

**Authors:** Yongsub Lim, Injae Yu, Dongmin Seo, U Kang, Lee Sael

**Affiliations:** 1 Data R&D Center, SK Telecom, Gyeonggi, Korea; 2 School of Computing, KAIST, Daejeon, Korea; 3 KISTI, Daejeon, Korea; 4 Department of Computer Science and Engineering, Seoul National University, Seoul, Korea

**Keywords:** Graph clustering, Markov clustering, Parallel clustering, Coarsening, Non-overlapping clusters, Protein complex finding

## Abstract

**Background:**

How can we obtain fast and high-quality clusters in genome scale bio-networks? Graph clustering is a powerful tool applied on bio-networks to solve various biological problems such as protein complexes detection, disease module detection, and gene function prediction. Especially, MCL (Markov Clustering) has been spotlighted due to its superior performance on bio-networks. MCL, however, is skewed towards finding a large number of very small clusters (size 1-3) and fails to detect many larger clusters (size 10+). To resolve this fragmentation problem, MLR-MCL (Multi-level Regularized MCL) has been developed. MLR-MCL still suffers from the fragmentation and, in cases, unrealistically large clusters are generated.

**Results:**

In this paper, we propose PS-MCL (Parallel Shotgun Coarsened MCL), a parallel graph clustering method outperforming MLR-MCL in terms of running time and cluster quality. PS-MCL adopts an efficient coarsening scheme, called SC (Shotgun Coarsening), to improve graph coarsening in MLR-MCL. SC allows merging multiple nodes at a time, which leads to improvement in quality, time and space usage. Also, PS-MCL parallelizes main operations used in MLR-MCL which includes matrix multiplication.

**Conclusions:**

Experiments show that PS-MCL dramatically alleviates the fragmentation problem, and outperforms MLR-MCL in quality and running time. We also show that the running time of PS-MCL is effectively reduced with parallelization.

## Background

Graph clustering is one of the most fundamental problems in graph mining and arises in various fields including bio-network analysis [1, 2]. Graph clustering is extensively studied and applied in protein complex finding, [3–5], disease module finding [6], and gene function prediction [7].

In general, the main task of the graph clustering problem is to divide the graph into cohesive clusters that have low interdependency: i.e., few inter-cluster edges and many intra-cluster edges. Additional domain-specific constraints can be added to the graph clustering to improve the clustering quality, however, we only focus on improving topology-based clustering as con-straints can be added easily afterward.

Among a number of clustering algorithms, MCL (Markov Clustering) [8] has received greatest attention in the bio-network analysis. Various studies have shown its superiority to other methods [3, 9–11]. However, MCL tends to result in too small clusters, which is called the fragmentation problem. Considering that many bio-network analysis related problems require cluster sizes in the range of 5–20 [12–14], fragmentation needs to be avoided. To solve the problem in MCL, R-MCL (Regularized-MCL) has been developed [15], but it often generates clusters that are too large, e.g., one cluster containing most of the nodes. Satuluri et al. [9] generalizes R-MCL to obtain clusters whose sizes are similar to the ones observed in real bio-networks by introducing a balancing factor. In the work, a large number of nodes belong to clusters of size 10–20 with an appropriate balancing factor; however, tiny clusters of size 1–3 also greatly increase compared with the original R-MCL. To improve the scalability of R-MCL, MLR-MCL (Multi-level R-MCL) has been developed [15]. MLR-MCL first coarsens a graph and then runs R-MCL with refinement. But, its coarsening scheme HEM (Heavy Edge Matching) is known to be inefficient for real world graphs, such as protein interaction networks, which have a heavy-tailed degree distribution [16–18].

In this paper, we propose PS-MCL (Parallel Shotgun Coarsened MCL), a parallel graph clustering method for bio-networks with an efficient graph coarsening scheme and parallelization. First, we propose SC (Shotgun Coarsening) scheme for MLR-MCL; SC allows grouping multiple nodes at a time [19]. Compared with HEM used in MLR-MCL, which is similar to a greedy algorithm for the traditional matching problem, SC coarsens a graph to have more cohesive super nodes. Moreover, the coarsened graph with a manageable size is obtained more quickly by SC than by HEM. Second, we carefully parallelize main operations in R-MCL which is a subroutine of MLR-MCL: i.e. *Regularize*, *Inflate* and *Prune* operations are parallelized. The latter two are column-wise operations by definition, and we parallelize them by assigning each column to a core. The former, *Regularize*, is a matrix multiplication. We divide matrix-matrix multiplication into a number of matrix-vector multiplications and parallelize them by distributing the vectors to multi-cores and sharing the matrix. Through experiments, we show that PS-MCL not only resolves the fragmentation problem but also outperforms MLR-MCL in quality and running time. Moreover, we show that PS-MCL gets effectively faster as more processing cores are used. PS-MCL produces clustering with the best quality, and its speed is comparable to MCL, which is a baseline method, and much faster than MLR-MCL, which is our main competitor (Table 1).

**Table 1:** Comparison of our proposed PS-MCL to MLR-MCL and MCL in clustering quality and speed

	(Proposed)		
	PS-MCL	MLR-MCL [15]	MCL [8]
Fragmentation	Least	Less	Most
Ncut	Small	Moderate	Large
Speed	Fast	Slow	Fastest

Note that PS-MCL produces clustering with the best quality, and its speed is comparable to MCL, which is a baseline method, and much faster than MLR-MCL, which is our main competitor

Our contributions are summarized as follows.

- **Coarsening**: We propose the Shotgun Coarsening (SC) scheme for MLR-MCL. SC allows merging multiple nodes to a super node at a time. Compared with the existing Heavy Edge Matching (HEM) coarsening method, SC improves both the quality and efficiency of coarsening.
- **Parallelization**: We carefully parallelize proposed algorithm using multiple cores by rearranging the operations to be calculable in a column-wise manner and assigning each column-wise computation to one core.
- **Performance**: Through experiments, we show that PS-MCL prefers clusters of sizes in the range of 10 to 20 and results in less fragmentation compared to MLR-MCL. We also show that PS-MCL is effectively parallelizable. As a consequence, PS-MCL outperforms MLR-MCL in both quality and speed (Table 1).

In the rest of the paper, we explain preliminaries including MCL based algorithms, describe our proposed method PS-MCL in detail, show experimental results on various protein interaction networks, and make a conclusion.

## Preliminaries

In this section, we explain existing MCL based algorithms: the original MCL, R-MCL, and MLR-MCL. Table 2 lists the symbols used in this paper.

**Table 2:** Table of symbols

Symbol	Description
*G*=(*V,E*)	Graph
*G*^′^=(*V*^′^,*E*^′^)	Coarsened graph
*n*	Number of nodes
*m*	Number of edges
*u,v*,*x,y*	Node
*S*	Super Node
*N* (*N*^′^)	Set of neighbors in *G* (*G*^′^)
*W* (*W*^′^)	Edge weight map in *G* (*G*^′^)
*A*	Adjacency matrix
*M,M*_*i*_,*M*_*G*_	Flow matrix
*M*_*G*_	Initial flow matrix
*r*	Regularization factor
*b*	Balancing factor
$\mathcal {C}$	Clustering

### Markov clustering (MCL)

MCL is a flow-based graph clustering algorithm. Let *G*=(*V,E*) be a graph with *n*=|*V*| and *m*=|*E*|, and *A* be the adjacency matrix of *G* where self-loops for all nodes are added. The (*i,j*)th element *M*_*ij*_ of the initial flow matrix *M* is defined as follows:

$$ M_{ij} = \frac{A_{ij}}{{\sum\nolimits}_{k=1}^{n} A_{kj}}. $$

Intuitively, *M*_*ij*_ can be understood as the transition probability or the amount of flow from *j* to *i*. MCL iteratively updates *M* until convergence, and each iteration consists of the following three steps.

- Expand: *M*←*M*×*M*.
- Inflate: $M_{ij} \leftarrow \left (M_{ij}\right)^{r} / {\sum \nolimits }_{k=1}^{n} \left (M_{kj}\right)^{r}$ where *r*>1.
- Prune: elements whose values are below a certain threshold are set to 0; every column is normalized to sum to 1.

When MCL converges, each column of *M* has at least one nonzero element. All nodes whose corresponding columns have a nonzero element in the same row are assigned to the same cluster. If a node has multiple nonzero elements in its column, a row is arbitrarily chosen. Although MCL is simple and intuitive, it lacks scalability due to the matrix multiplication in the expanding step, and outputs a large number of too small clusters, e.g., outputs 1416 clusters from a network with 4741 nodes (fragmentation problem) [15].

### Regularized-MCL (R-MCL)

One reason of the fragmentation of clusters in MCL is that the adjacency structure of a given graph is used only at the beginning, which leads to diverging columns for neighboring node pairs. To resolve this fragmentation problem, R-MCL [15] regularizes a flow matrix instead of expanding it. The flow of a node is updated by minimizing the weighted sum of KL divergences between the target node and its neighbors. This minimization problem has a closed form solution, and consequently, the regularizing step of R-MCL is derived as follows.

- Regularize *M*=*M*×*M*_*G*_, where *M*_*G*_ is an initial flow matrix defined as *M*_*G*_=*AD*^−1^, and *A* is the adjacency matrix of *G* with the added self-loops and the weight transformation [15], and *D* is the diagonal matrix from *A* (i.e., $D_{ii} = {\sum \nolimits }_{k=1}^{n} A_{ik} $).

R-MCL finds a smaller number of clusters than MCL does.

A problem of R-MCL is that it finds clusters whose sizes are spread over a wide range, while clusters in bio-networks usually are in the size range of 5–20 [12, 13]. To resolve the problem, [9] generalizes the regularization step as follows:

- $mass(i) = {\sum \nolimits }_{j=1}^{n} M_{ij}$.
- *M*_*R*_=column_normal (*diag*(*M*^⊤^×*mass*)^−*b*^×*M*_*G*_), where *b* is a balancing parameter.
- Regularized by *M*=*M*×*M*_*R*_.

The balancing parameter *b* controls the degree of balances in the cluster sizes; higher *b* encourages more balanced clustering. The intuition of this generalization is to penalize flows to a node currently having a large number of incoming flows. Note that *b*=0 is equal to the original R-MCL.

### Multi-level R-MCL (MLR-MCL)

MLR-MCL uses graph coarsening to further improve the quality and the running time of R-MCL [15]. Graph coarsening means to merge *related* nodes to a super node. MLR-MCL first generates a sequence of coarsened graphs: (*G*_0_,*G*_1_,…,*G*_*ℓ*_) where *G*_0_ is the original graph and *G*_*ℓ*_ is the most coarsened (smallest) graph. For *i*=*ℓ* down to 1, R-MCL is run on *G*_*i*_ only for a few iterations, and the computed flows on *G*_*i*_ are projected to *G*_*i*−1_. After reaching the original graph *G*_0_, R-MCL is run until convergence. Algorithm 1 shows the overall procedure of MLR-MCL. Although the description is for *b*=0,*b*>0 can be also used by changing *M*_*G*_ to *M*_*R*_ as defined in the previous section.

The original R-MCL and MLR-MCL use HEM (Heavy Edge Matching), which picks an unmatched neighbor connected to the heaviest edge for a given node, to coarse the graph [15]. In HEM, the node *v* to which a node *u* is merged is determined as follows:

$$ v = \text{arg\,max}_{v'\in N_{unmatched}(u)} W\left(u,v'\right), $$

where *N*_*unmatched*_(*u*) is the set of unmatched neighbors of *u*, and *W*(*u,v*^′^) is the weight between *u* and *v*^′^. Note that HEM allows a node to be matched with at most one other node. MLR-MCL assigns all flows of a super node to one of its children for the flow projection. It is shown that a clustering result is invariant on the choice of the child to which all flows are assigned. For more details, refer to [15]. Note that MLR-MCL greatly reduces the overall computation of R-MCL since the flow update is done for the coarsened graph which is smaller than the original graph.

**Limitation of HEM.** HEM of MLR-MCL has two main limitations. First, the strategy of HEM that merges at most two single nodes can lead to undesirable coarsening where super nodes are not cohesive enough (see “Cohesive super node” section for details). Second, HEM is known to be unsuitable for real-world graphs [19] due to skewed degree distribution of the graphs which prevents the graph size from being effectively reduced (see “Quickly reduced graph” section for details). These shortages of HEM make MLR-MCL inefficient for real-world graphs. To overcome this, in the next section we propose PS-MCL that allows multiple nodes to be merged at a time.


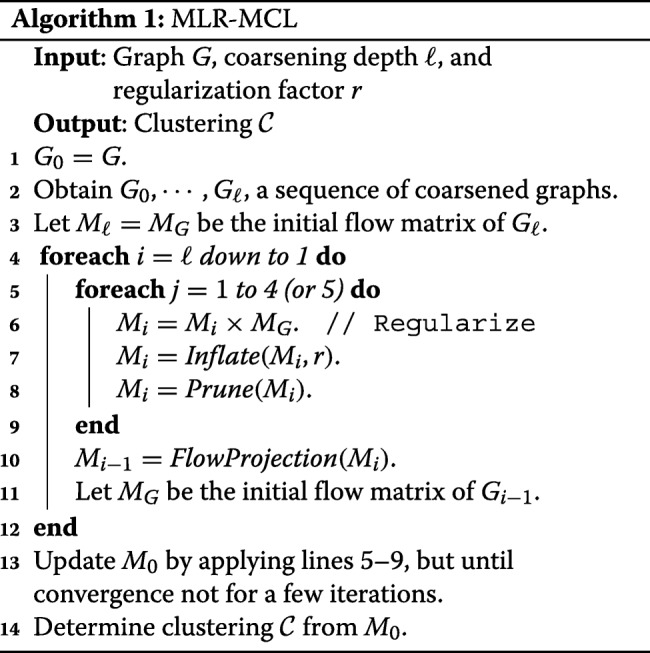


## Implementation

In this section, we describe our proposed method PS-MCL (Parallel Shotgun Coarsened MCL) which improves MLR-MCL in two perspectives: 1. increasing efficiency of the graph coarsening and 2. parallelizing the operations of R-MCL.

### Shotgun coarsening (SC)


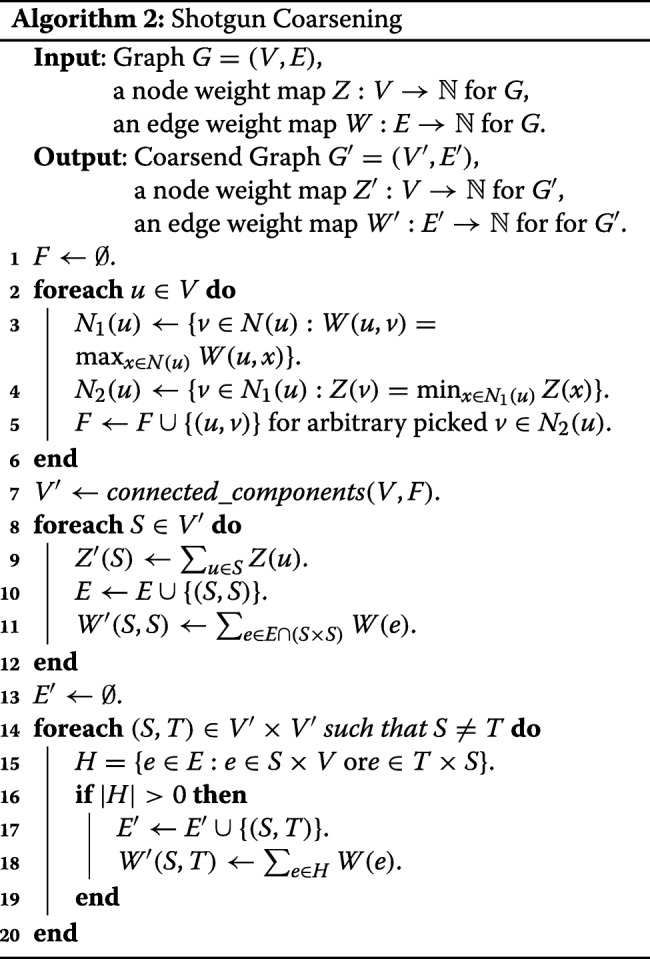


As described previously, HEM is ineffective on real world graphs. To overcome the limitation of HEM, we propose to use a graph coarsening which allows merging multiple nodes at a time. We call this scheme Shotgun Coarsening (SC) because it aggregates satellite nodes to the center one. Algorithm 2 describes the proposed SC coarsening method where *N*(*u*) denotes a set of neighbors of *u* in *G*=(*V,E*), and *connected*_ *components*(*V,F*) outputs a set of connected components, each of which is a set of nodes, of the graph (*V,F*).

Our SC algorithm consists of three steps: 1) identify a set *F* of edges whose end nodes will be merged (lines 1–6), 2) determine a set *V*^′^ of super nodes of a coarsened graph and associated weights to them (lines 7–12), and 3) determine a set *E*^′^ of edges between super nodes and their weights (lines 13–20). Let *G*=(*V,E*) be an input graph to be coarsened, $Z:V\rightarrow \mathbb {N}$ be a node weight map for *G*, and $W:E\rightarrow \mathbb {N}$ be an edge weight map for *G*. In the first step, we visit every node of *G* in an arbitrary order (line 2), and for each node *u*∈*V* visited, we find the best match node *v*. Precisely, the algorithm finds the neighboring node of *u* with the highest edge weight to *u* (line 3), i.e.,

$$ v = \text{arg\,max}_{x\in N(u)} W(u,x). $$

There may be multiple neighbors with the same highest weight. Let *N*_1_(*u*) be the set of those neighbors; then, in this case, the one with the smallest node weight is chosen among them (line 4), i.e.

$$ v = \text{arg\,min}_{x\in N_{1}(u)} Z(x). $$

This strategy of preferring a smaller node weight at the same edge weight prevents the emergence of an over-coarsened graph containing an excessively massive super node. Note that if every node in an initial graph has weight 1, the weight of a super node in a coarsened graph is equal to the number of nodes merged to create that super node. If there are multiple neighbors with the same highest edge weight and the smallest node weight, any *v* is arbitrarily chosen among the ties. Edge (*u,v*) is added to *F* (line 5).

The second step determines super nodes and associated weights to them. Note that for (*u,v*)∈*F*, *u* and *v* should belong to the same super node by definition. By mathematical induction, two nodes belong to the same super node if and only if they are reachable along edges in *F*. As a result, we can identify a set *V*^′^ of super nodes by computing connected components of the graph (*V,F*) (line 7).

After finding *V*^′^, we determine weights of super nodes in *V*^′^ and their self-loops as follows. For each super node *S*∈*V*^′^, its node weight *Z*^′^(*S*) is defined by the sum of weights of nodes in *V* that belong to *S* (line 9). The self-edge (*S,S*) is added to *E*^′^ (line 10) and its weight *W*^′^(*S,S*) is defined by the sum of weights of edges in *E* whose end nodes belong to *S* (line 11).

The last step determines non-self edges between nodes in *V*^′^ and their edge weights as follows. For each unordered pair (*S,T*)∈*V*^′^×*V*^′^, find a set *H* of edges in *E* that one end node is in *S* and the other is in *T* (line 15). If *H*≠*∅* (line 16), (*S,T*) is added to *E*^′^ (line 17), and *W*^′^(*S,T*) is defined by the sum of weights of edges in *H* (line 18). Otherwise, there is no edge between *S* and *T*.

**Skip Rate** In practice, a graph can be reduced too quickly by SC if it has *super-hub* nodes. To coarsen the graph to a reasonable size, we propose to randomly skip merging while iterating nodes in SC, i.e. with probability 0≤*p*<1, lines 3–5 are not executed. We call *p* a skip rate, and use *p*=0.5 in this paper.

#### Cohesive super node

**Fig. 1:**
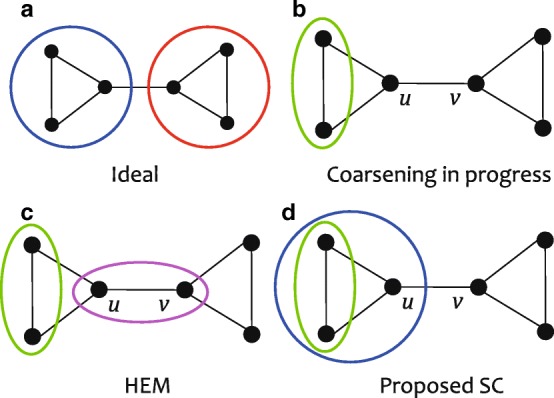
Effectiveness of our proposed SC method compared with HEM. **a** Ideal coarsening for the graph. **b** Coarsening in progress. For the first merging, the leftmost two nodes are chosen. **c** For the second node to be merged, *u* is chosen. Since *v* is the only candidate for merging in HEM, *u* and *v* are merged to a super node. **d** In SC, the green super node is also a candidate for *u*. Since the weight of *u* to the green node is larger than that to *v*, *u* is merged to the green super node

The goal of coarsening is to merge tightly connected nodes to one super node. In this aspect, HEM may prevent a super node from being cohesive. Figure 1a shows the ideal coarsening for a given graph. Let us assume that for the first merging, the leftmost two nodes are merged as shown in Fig. 1b, and the next node to be merged is *u*. If we use HEM, *v* is chosen since it is the only candidate, leading to Fig. 1c. Note that although *u* has more edges to the green super node than to *v*, it should be merged with *v*. Obviously, the result is undesired for good coarsening. In contrast, SC (Fig. 1d) chooses the green super node for *u* since the weight to the green node is larger than that to *v*. As a result, SC generates more cohesive super nodes than those by HEM, leading to a high quality coarsened graph.

#### Quickly reduced graph

**Fig. 2:**
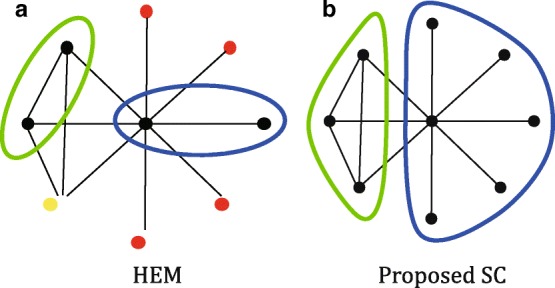
Efficiency of our proposed coarsening method SC compared with HEM. **a** Result of one coarsening step by HEM. The red and yellow nodes are eventually merged to the blue and green nodes, respectively, but it takes 5 more coarsening steps. **b** Result of one coarsening step by SC. The result is the same as (**a**) after 5 more coarsening

Ideally, at each step of the coarsening, the number of nodes should halve; but that does not happen for real world graphs due to their highly skewed degree distribution [19]. In other words, a large number of coarsening steps are needed to obtain a coarsened graph of a manageable size, leading to large memory spaces for storing graphs themselves and node merging information. This problem arises mainly due to star-like structures, which is depicted in Fig. 2a. The red and yellow nodes are eventually merged with the blue and the green groups, respectively, but it needs 5 more coarsening steps because only two nodes can be merged. Note that for an additional coarsening step, we need spaces to store one graph and mapping from a node to a super node; if the graph size is not effectively reduced, the amount of the required spaces greatly increases with the coarsening depth. This inefficiency can be resolved by SC as shown in Fig. 2b. In contrast that 5 more coarsening steps are required with HEM, only one step is enough in SC.

### Parallelization

We also improve MLR-MCL via multi-core paralle-lization for its three main operations: *Regularize*, *Inflate*, and *Prune*. For *Regularize*, we parallelize the computation by assigning columns of the resulting matrix into cores. In other words, for *M*_3_=*M*_1_×*M*_2_, we divide the computation as follows.

$$ M_{3}(,i) = M_{1} \times M_{2}(,i), $$

where *M*_*k*_(,*i*) denotes the *i*-th column of *M*_*k*_. Com-puting *i*th column of *M*_3_ is independent of computing other columns *j*≠*i* of *M*_3_, and thus we distribute the columns of *M*_2_ to multiple cores while keeping *M*_1_ in a shared memory. *Inflate* and *Prune* themselves are columnwise operations. Thus, the computation on each column is assigned to a core.

**Fig. 3:**
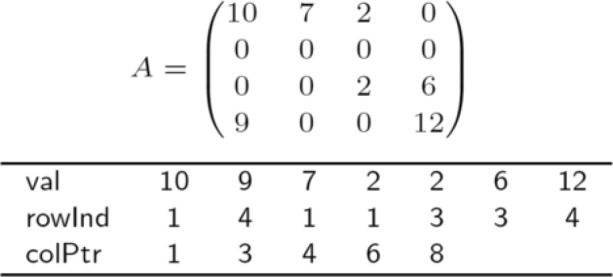
CSC format for sparse matrix representation. The top figure is a given matrix *A*, and the bottom one is the corresponding CSC representation of *A*. As the size of matrix gets larger and the matrix gets sparser, CSC gets much efficient since the space complexity of CSC is proportional to the number of nonzero elements, not to the matrix size


For efficiency in memory usage, we use the CSC (Compressed Sparse Column) format [20] to represent a matrix, which requires much less memory when storing a sparse matrix compared to a two-dimensional array format. In essence, the CSC format only stores nonzero values of a matrix. Note that this strategy is efficient especially for real world graphs which are very sparse in general, e.g. |*E*|=*O*(|*V*|). Figure 3 shows the CSC format for an example matrix. To access the nonzero elements from the *j*th column (1-base indexing), we 1) obtain *a*=colPtr[*j*] and *b*=colPtr[*j*+1]−1 and 2) for *a*≤*i*≤*b*, get val[*i*]=*A*(rowInd[*i*],*j*). For example, to access the first column, we first obtain *a*=1 and *b*=2. By checking val[*i*] and rowInd[*i*] for 1≤*i*≤2, we identify the two nonzero values 10 and 9 at the first and the fourth rows, respectively, in the first column: i.e., *A*(1,1)=10 and *A*(4,1)=9 since val[1]=10 with rowInd[1]=1 and val[2]=9 with rowInd[2]=4.

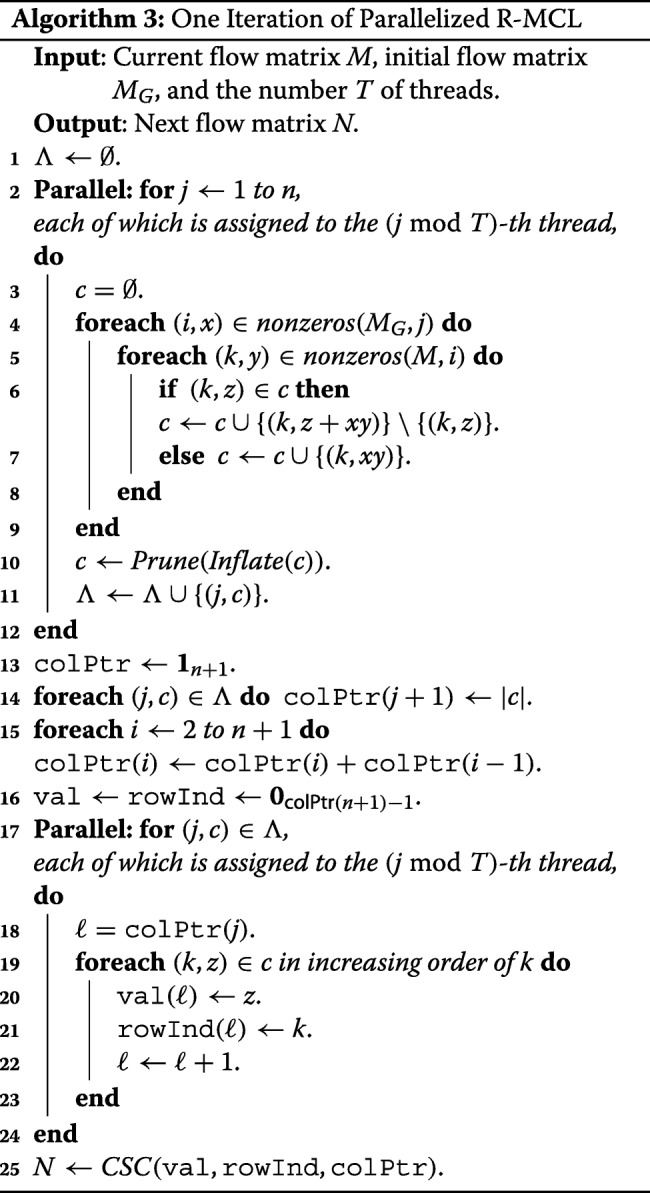


Algorithm 3 shows our implementation for one iteration of parallelized R-MCL with the CSC format. In the algorithm, *nonzeros*(*M,j*) is a set of pairs (*i,x*) indicating nonzeros in the *j*-th column of the matrix *M*, i.e., *M*(*i,j*)=*x*; **0**_*n*_ and **1**_*n*_ denote *n* dimensional vectors all of whose elements are 0 and 1, respectively. Lines 4 to 9 correspond to *Regularize*. Each thread running in a dedicated core performs *c*=*M*×*M*_*G*_(,*j*) for each column *j* assigned to it, and as a result, (*j,c*) is added to *Λ* after applying *Inflate* and *Prune* to *c*. Note that although we do not describe *Inflate* and *Prune* in Line 10, its implementation is trivial for each column *c*.

Lines 13 to 16 correspond to constructing colPtr, and allocating spaces for val and rowInd for the resulting matrix *N*, which is done sequentially. After-wards, Lines 17 to 24 correspond to filling val and rowInd using *Λ* and colPtr in parallel on the columns. Note that the positions of val and rowInd to be updated for each column are specified in colPtr, and they do not overlap.

## Results

We present experimental results to answer the following questions.

- Q1 How does PS-MCL improve the distribution of cluster sizes compared with MLR-MCL?
- Q2 What is the performance of PS-MCL compared with MLR-MCL in quality and running time?
- Q3 How much speedup do we obtain by parallelizing PS-MCL?
- Q4 How accurate are clusters found by PS-MCL compared to the ground-truth?

Table 3 lists the used datasets in our experiments. We use various bio-networks for evaluating the clustering quality and the running time; the largest dataset DBLP is used for scalability experiments.

**Table 3:** Dataset description used in our experiments

Name	Nodes	Edges	Density (10^−4^)	Description
BioPlex [23]	10,961	56,553	9.42	PPI networks of Homo sapiens in the BioPlex 2.0 Network
DIP-droso [21]	22,595	69,148	2.71	PPI network of Drosophila melanogaster
Drosophila [24]	6600	19,820	9.1	PPI network of Drosophila melanogaster
MINT [25]	3872	56,937	75.97	combined PPI network of 325 different organisms
Yeast-1 [26]	2353	36,790	132.95	Genome-scale epistasis map of Schizosaccharomyces pombe
Yeast-2 [27]	2428	4606	15.63	PPI network of Saccharomyces cerevisiae
Yeast-3 [28]	3886	57,782	76.55	PPI network of Saccharomyces cerevisiae
Yeast-4 [29]	2223	7049	28.54	PPI network of Saccharomyces cerevisiae
DIP-yeast [21]	4929	17,786	14.64	PPI network of Saccharomyces cerevisiae
BioGRID-homo [22]	20,837	288,224	13.28	PPI network of Homo sapiens
DBLP	317,080	1049,866	0.21	Coauthorship network

### Experimental settings

**Machine.** All experiments are conducted on a work-station with double CPU Intel(R) Xeon(R) CPU E5-2630 v4 @ 2.20GHz and 250GB memory.

**Evaluation criteria.** To evaluate the quality of clustering $\mathcal {C}$ for Q1 and Q2, we use the average NCut [15] defined as follows.

$$ AverageNCut(\mathcal{C}) = \frac{1}{|\mathcal{C}|}\sum\limits_{c\in \mathcal{C}} NCut(c), $$

where

$$ NCut(c) = \frac{{\sum\nolimits}_{u\in c, v\notin c} A(u,v)} {{\sum\nolimits}_{u\in c} degree(u)}. $$

For answering Q4, we focus on protein complex finding problem and use the accuracy measure defined by Hernandez et al. [4] as follows. Let $\mathcal {G}$ be the set of ground truth clusters (protein complexes); then the degree of overlap *T*_*gc*_ for every $g\in \mathcal {G}$ and $c\in \mathcal {C}$ is defined as:

$$ T_{gc} = |g\cap c|. $$

The accuracy *ACC* is the geometric mean of Sensitivity *SST* and Positive Predictive Value *PPV*:

$$\begin{array}{*{20}l} SST =& \frac{{\sum\nolimits}_{g\in \mathcal{G}} max_{c\in\mathcal{C}}{T_{gc}}} {{\sum\nolimits}_{g\in |\mathcal{G}|} |g|} \\ PPV =& \frac{{\sum\nolimits}_{c\in \mathcal{C}} max_{g\in \mathcal{G}}{T_{gc}}}{{\sum\nolimits}_{c\in \mathcal{C}} |c|} \\ ACC =& (SST \times PPV)^{\frac{1}{2}} \end{array} $$

**Parameter.** We use the coarsening depth of 3 for PS-MCL and MLR-MCL with which the improvement in quality and speed is large while the number of resulting clusters remains reasonable.

### Performance of SC

**Fig. 4:**
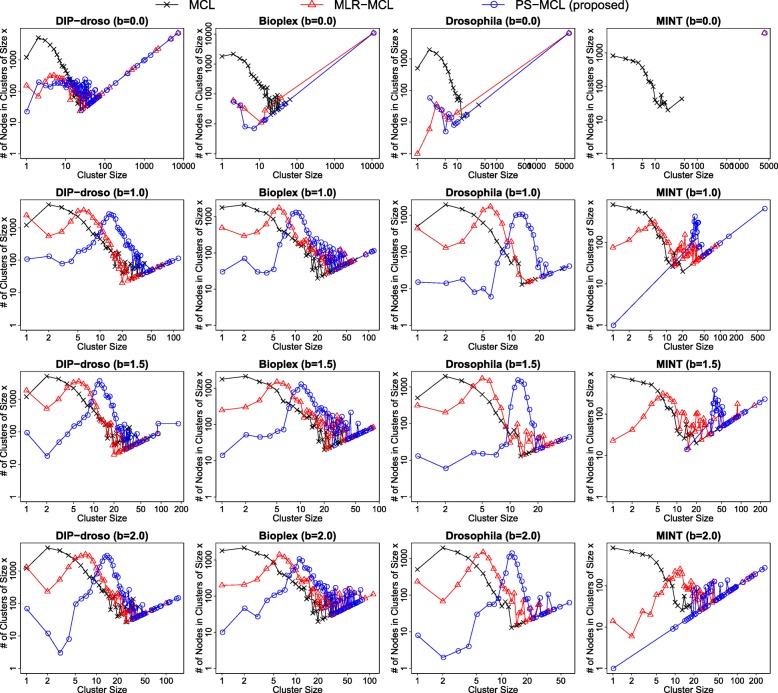
The number of nodes belonging to clusters of specific sizes. With balancing factor *b*=0, i.e. without considering balance, PS-MCL and MLR-MCL do not find clusters properly. In general, they output too large clusters and even group all the nodes into one cluster for MINT. Using balancing factor *b*>0, both result in clusters whose sizes are concentrated around a small value. That value is larger in PS-MCL than in MLR-MCL. Observe that MLR-MCL makes a significantly large number of tiny clusters including singletons which are meaningless in clustering. In contrast, our proposed PS-MCL greatly reduces that number: less than 5% compared with the number by MLR-MCL. For all cases, MCL suffers from the cluster fragmentation problem. For the other datasets in 3, we observe the same patterns described here

In this section, we answer Q1 and Q2. Figure 4 shows comparison of PS-MCL with MLR-MCL and MCL. The horizontal axis denotes the cluster size, and the vertical axis denotes the number of nodes belonging to a specific cluster size.

Before going into details, we briefly summarize the results of MCL which are invariant within each column of Fig. 4 because of the lack of the balancing concept in MCL. As discussed in [15], for all cases, MCL suffers from the fragmentation problem that a large portion of nodes belongs to very tiny clusters of size 1–3. We provide results of MCL as a baseline in the figure, and the following analysis focuses on comparing the MLR-MCL and PS-MCL.

For all of our datasets, we observe the same patterns described in the following Observations 1–3, though we present the four representative results in the Fig. 4.

#### **Observation 1**

(Too massive cluster without balancing) Without balancing, i.e. *b*=0, a large number of nodes are assigned to one cluster. Often, the entire graph becomes one cluster. Balancing factor of 1.0 to 1.5 resulted in reasonable cluster size distribution for most of the networks.

**Fig. 5:**
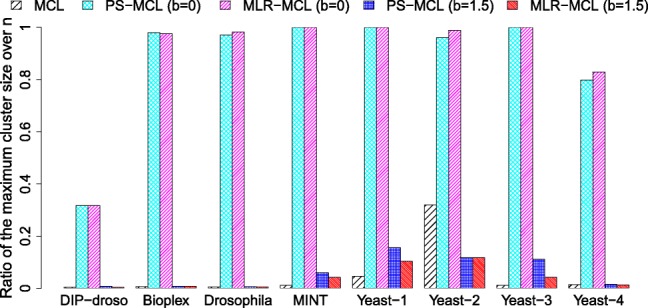
Ratio of the largest cluster size over the number of nodes. Without balancing, i.e. *b*=0, PS-MCL and MLR-MCL group too many nodes to one cluster

The first row of Fig. 4 corresponds to the result without balancing, i.e. *b*=0. In this case, both PS-MCL and MLR-MCL group too many nodes into one cluster. Especially, on MINT, both output only one cluster containing all nodes in the graph. Figure 5 shows the ratio of the largest cluster size over the number of nodes for the bio-networks listed in 3. Note that the largest cluster sizes for BioPlex, Drosophila, MINT, Yeast-1, Yeast-2, and Yeast-3 are nearly the same as the total number of nodes; those for DIP-droso and Yeast-4 are relatively smaller, but still, occupy a large proportion in the entire size.

#### **Observation 2**

(PS-MCL preferring larger clusters than MLR-MCL) With *b*>0, the cluster size with the maximum number of total nodes in PS-MCL is larger than that in MLR-MCL.

The second, third and fourth rows of Fig. 4 show the results of varying the balancing factor *b*∈{1,1.5,2}, respectively. In contrast to the case of *b*=0, the cluster sizes of PS-MCL and MLR-MCL are concentrated on certain sizes. The mode of cluster size in PS-MCL is larger than that in MLR-MCL. The modes in PS-MCL are 10–20 for DIP-droso, BioPlex and Drosophila, and 20–50 for MINT; those in MLR-MCL are 5–10 for all. This observation is useful in practice when we want to cluster at a certain scale.

#### **Observation 3**

(PS-MCL with less fragmentation than MLR-MCL) With *b*>0, PS-MCL results in a significantly smaller number of fragmented clusters whose sizes are 1–3 compared with MLR-MCL.

PS-MCL achieves concentrated cluster sizes as well as avoids the fragmented clusters; MLR-MCL and MCL still suffer from the fragmentation. The number of nodes belonging to very small clusters in PS-MCL is much smaller than that in MLR-MCL. For instance, the number of nodes belonging to clusters of size 1–3 in PS-MCL is less than 5% of that in MLR-MCL for the DIP with *b*=1.5.

#### **Observation 4**

(PS-MCL better than MLR-MCL in time and NCut) PS-MCL results in a faster running time with a smaller NCut than MLR-MCL does.

**Fig. 6:**
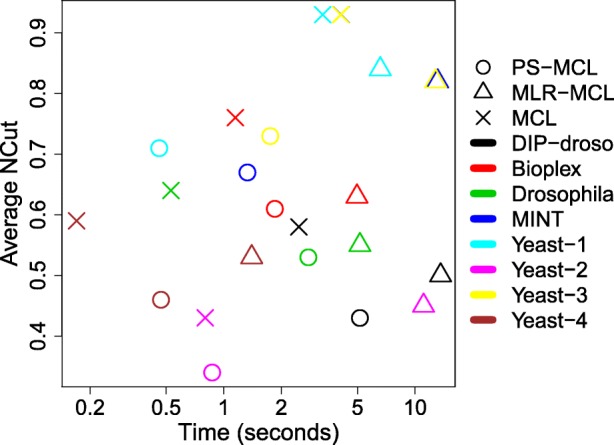
Time vs. average NCut for all datasets with *b*=1.5 for PS-MCL, MLR-MCL and MCL. We use 4 cores for PS-MCL. PS-MCL was 21% faster in running time and had 87% better NCut on average over all datasets compared with MLR-MCL. Although MCL is faster than PS-MCL for some networks, its average NCut is higher than that by PS-MCL for all networks

Figure 6 shows the plot of running time versus the average NCut. PS-MCL runs faster, down to 21%, and outputs clustering with a smaller average NCut, down to 87%, than MLR-MCL does on average. For some cases, MCL is faster than PS-MCL, but for all cases, its average NCut is worse than that by PS-MCL.

### Performance of parallelization

**Fig. 7:**
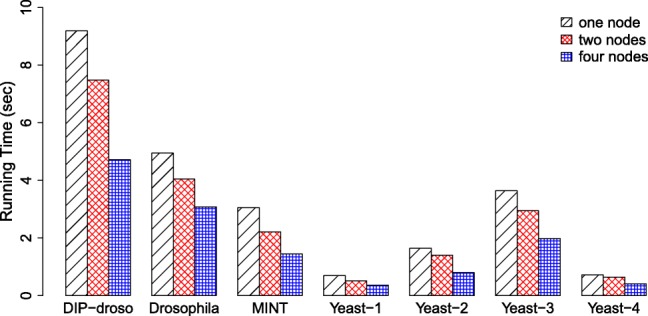
Running time of PS-MCL for bio-networks in 3 while increasing the number of cores. Note that the running time is effectively reduced as the number of cores increases

In this section, we answer Q3. We use *b*=1.5 for PS-MCL and MLR-MCL. Figure 7 shows the performance evaluation results for PS-MCL on the bio-networks in 3 with increasing cores. For all cases, PS-MCL gets faster as the number of cores increases.

**Fig. 8:**
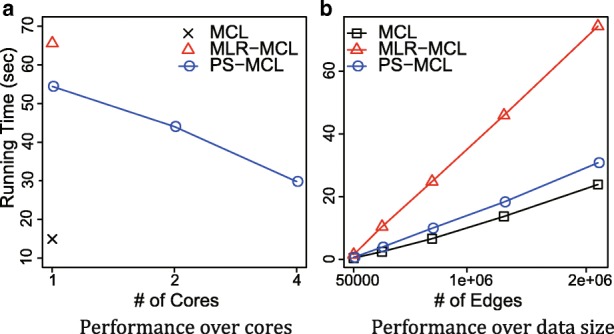
Running time of PS-MCL, MLR-MCL, and MCL. **a** Running time on DBLP while varying the number of cores. Running time of PS-MCL is improved down to 81% and 55% with 2 and 4 cores, respectively, compared that with a single core. Furthermore, the single core performance of PS-MCL outperforms MLR-MCL. **b** Running time on DBLP while varying data sizes. Running times of all methods are linear on the number of edges in the graphs, and PS-MCL outperforms MLR-MCL for all cases

To test the scalability more effectively, we use DBLP, the largest in our datasets though it is not a bio-network. Figure 8a shows the speed up of PS-MCL while increasing the number of cores, compared with MLR-MCL and MCL. We use points, not lines, for MLR-MCL and MCL since they are single-core algorithms. PS-MCL outperforms MLR-MCL regardless of the number of cores and becomes faster effectively as the number of cores increases. Precisely, the running time of PS-MCL is improved down to 81% and 55% with 2 and 4 cores, respectively, compared that with a single core.

Figure 8b shows the running time of PS-MCL while increasing data sizes, compared with MLR-MCL and MCL. Here, we use 4 cores for PS-MCL. To obtain various sizes of graphs, we first take principal submatrices from the adjacency matrix of DBLP with sizes {20*%*,40*%*,60*%*,80*%*,100*%*} of the total, and use the giant connected components of them. As shown in the figure, the running times of all the methods are linear on the graph sizes, and PS-MCL outperforms MLR-MCL for all scales. Note that although MCL is slightly faster than PS-MCL, MCL has fragmentation problem and worse NCut while PS-MCL has no fragmentation problem and better NCut, as shown in Figs. 4 and 6.

### Protein complex identification

In this section, we use the two bio-networks, i.e., DIP-yeast [21] and BioGRID-homo [22] described in 3, to answer Q4 on protein complex finding problem. The ground-truth protein complexes information are extracted from CYC2008 2.0 [13] for DIP-yeast and CORUM [14] for BioGRID-homo. The complexes are used as reference clusters for measuring the accuracy.

**Fig. 9:**
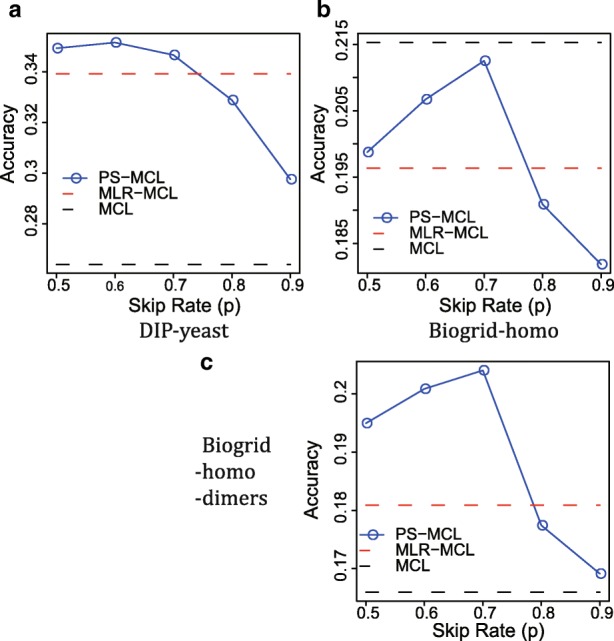
Accuracy of PS-MCL with varying skip rates, MLR-MCL, and MCL. The performance of PS-MCL varies depending on *p*, but is better than that of MLR-MCL in the range of 0.5≤*p*≤0.7. Comparing to MCL, PS-MCL is slightly less accurate. But, we observe that it is due to many dimer structures, and excluding dimers, PS-MCL greatly outperforms MCL (**c**). **a** DIP-yeast **b** Biogrid-homo **c** Biogrid-homo-dimers

Figure 9 shows the performance of PS-MCL while varying skip rates, in comparison with MLR-MCL and MCL (Note: The skip rate is not applicable to MLR-MCL and MCL, leading to one accuracy value. For clear performance comparison, we represent that value by the horizontal dash line along the *x*-axis.). Remind that the skip rate *p* determines the chance that each node is skipped and thus not merged with others. Namely, the smaller *p*, the more aggressive coarsening. In the figure, PS-MCL performs the best with moderate values of *p*—0.6 and 0.7 for DIP-yeast and BioGRID-homo, respectively. For both networks, PS-MCL consistently outperforms MLR-MCL with 0.5≤*p*≤0.7: the accuracy of PS-MCL is higher than that of MLR-MCL up to 3.66*%* for DIP-yeast and 8.24*%* for BioGRID-homo. This result makes sense because SC with too large *p* hardly reduces a graph in size, while too small *p* leads to too large clusters due to aggressive coarsening.

PS-MCL achieves up to 33.2*%* higher accuracy than MCL for DIP-yeast, and 98.7*%* of the MCL accuracy for BioGRID-homo. This is due to many dimer structures present in the CORUM database. Exclusion of dimers in the database, PS-MCL greatly outperforms MCL as shown in Fig. 9c. Although PS-MCL is not effective in finding dimers, note that MCL suffers from the fragmentation problem (Fig. 4) and performs poorly in internal evaluation by Average NCut (Fig. 6) which assesses the potentials of finding well-formed but undiscovered clusters.

## Conclusion

In this paper, we propose PS-MCL, a parallel graph clustering method which gives superior performance in bio-networks. PS-MCL includes two enhancements compared to previous methods. First, PS-MCL incor-porates a newly proposed coarsening scheme we call SC to resolve the inefficiency of MLR-MCL in real-world networks. SC allows merging multiple nodes at a time, leading to reducing the graph size more quickly and making super nodes much cohesive than HEM used in MLR-MCL. Second, PS-MCL gives a multi-core parallel algorithm for clustering to increase scalability. Extensive experiments show that PS-MCL results in clusters that generally have larger sizes than those by MLR-MCL, and also greatly alleviate the fragmentation problem. Moreover, PS-MCL finds clusters whose quality is better than those by MLR-MCL in both internal (average NCut) and external (reference clusters) criteria. Also, as more cores are used, PS-MCL gets faster and outperforms MLR-MCL in speed even with a single core.

The PS-MCL‘s capability to quickly find mid-size clusters in large scale bio-networks has wide range of applicability on systems biology. Although we have only shown that PS-MCL effectively find mid-size protein complexes on two protein-protein interaction network compared to existing topology-based clustering algorithms, we believe that it can be effectively applied on function prediction, disease modules detection, and other systems biology analysis.

## Availability and requirements

**Project name:** PS-MCL;

**Project home page:**https://github.com/leesael/PS-MCL;

**Operating system(s):** Platform independent (tested on Ubuntu);

**Programming language:** Java;

**Other requirements:** Java 1.8 or higher;

**License:** BSD Any restrictions to use by non-academics: licence needed.

## Abbreviations

HEM: Heavy edge matching
MCL: Markov clustering
MLR-MCL: Multi-level R-MCL
PS-MCL: Parallel shotgun coarsening MCL
R-MCL: Regularized-MCL
SC: Shotgun coarsening
